# Effect of infection of potato plants by Potato virus Y (PVY), Potato virus S (PVS), and Potato virus M (PVM) on content and physicochemical properties of tuber starch

**DOI:** 10.1002/fsn3.3386

**Published:** 2023-05-07

**Authors:** Gulnazym Ospankulova, Vadim Khassanov, Svetlana Kamanova, Dana Toimbayeva, Saule Saduakhasova, Berdibek Bulashev, Gulnar Aidarkhanova, Yernaz Yermekov, Linara Murat, Bakhyt Shaimenova, Marat Muratkhan, Wenhao Li

**Affiliations:** ^1^ Department of Food Technology and Processing Products, Technical Faculty Saken Seifullin Kazakh Agrotechnical University Nur‐Sultan Kazakhstan; ^2^ Department of Biology, Plant Protection and Quarantine, Agronomic Faculty Saken Seifullin Kazakh Agrotechnical University Astana Kazakhstan; ^3^ Engineering Research Center of Grain and Oil Functionalized Processing in Universities of Shaanxi Province, College of Food Science and Engineering Northwest A&F Yangling China; ^4^ Department of Food Production Technology and Biotechnology, The Engineering–Technological Faculty Shakarim University Semey Kazakhstan

**Keywords:** physicochemical properties, potato tubers, potato virus, starch

## Abstract

Potato virus Y (PVY), Potato virus S (PVS), and Potato virus M (PVM) infection of potato plants leads to decreased dry matter and starch content in tubers. Starch samples from potato tubers infected with PVY, PVS, and PVM had higher amylose content. Granules of starch isolated from potato tubers infected by PVS exhibit larger granules than starch granules isolated from tubers of healthy plants. In contrast, in the case of PVM and PVY infection, starch granules were significantly smaller in diameter. A decrease in the degree of crystallinity has been observed in all samples of starches obtained from the tubers of infected plants compared to starch isolated from tubers of healthy plants. A slight decrease in gelatinization temperature was noted for starch samples isolated from tubers infected by PVY and PVM, and a slight increase in gelatinization temperature for starch samples isolated from tubers infected by PVS compared to starch isolated from tubers of healthy plants. In all samples of starch obtained from tubers of infected plants, an increase in the value of gelatinization enthalpy was observed. Thus, it can be concluded that damage to potato plants by PVM and PVY leads to a significant decrease in the quality of starch in tubers. At the same time, infection by PVS had practically no considerable effect.

## Graphical abstract

Damage to potato plants by potato viruses M and Y leads to a significant decrease in the quality of starch in tubers. At the same time, infection by Potato virus S had practically no considerable effect.

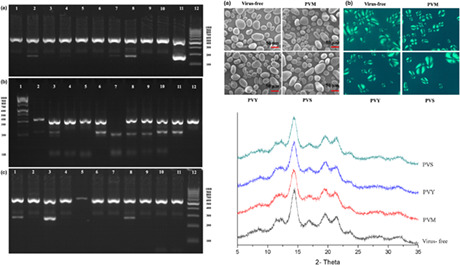

## 1 INTRODUCTION

Among the plethora of diseases affecting potato plants that viral etiology is the most common and causes significant damage to seed and tuber production. According to numerous sources, there are 40–50 viruses that infect potato plants all over the world (Bashir et al., 2021; Kreuze et al., 2020; Loebenstein et al., 2001; Valkonen, 2007; Wang et al., 2011), of which the following are considered the main significant potato viruses: Potato leafroll virus, Luteovirus (PLRV), Potato virus A, Potyvirus (PVA), Potato virus Y, Potyvirus (PVY), Potato virus X, Potexvirus (PVX), Potato virus S, Carlavirus (PVS), and Potato virus M, Carlavirus (PVM) (Bai et al., 2007; Rogozina et al., 2019; Valkonen, 2015; Wang et al., 2011; Zhang et al., 2010).

Viral infection of potato plants affects both the yield and quality of potatoes (Kotzampigikis et al., 2008). Contemporary research suggests that the loss of yield associated with viral infection of potato plants varies from 10 to 80% (Awasthi & Verma, 2017; Bashir et al., 2021; Hao et al., 2007; Huang et al., 2009; Kumar et al., 2020; Kumar et al., 2019; Wang et al., 2011; Wang et al., 2005; Whitworth et al., 2006). Most damaging effects are observed in the plants infected by PLRV and PVY (Kotzampigikis et al., 2008; Weidemann, 1988). PLRV and PVY infection have a fast rate of spread by vectors such as aphids (Gray et al., 2010), which explains their highly damaging effect on potatoes causing severe and moderate symptoms in plants (leaf curl, wrinkle and stripe mosaics, and leaf mosaic curl) that, if plants are propagated vegetatively, will almost always infect the propagated plants through tubers. Losses in yields after a combined infection by viruses can be even more severe (Struik & Wiersema, 1999).

Viruses, being obligate parasites and affecting the metabolism of plants, cause a violation of physiological processes, resulting in a decrease not only in the productivity of plants but also affecting the biochemical composition of tubers. As a result, plants grown from previously infected seed tubers (secondary infection) usually produce tubers unsuitable for sale (Bashir et al., 2021).

Infection by plant viruses leads to biochemical and physiological changes in photosynthesis, CO_2_ assimilation, and starch accumulation (Trethewey & Smith, 2000). It is noted that viruses usually suppress the accumulation of starch in the leaves of plants at an early stage of infection (Zhao et al., 2022). Infection of potato plants with PLRV, PVY, PVS, and PVM leads to a decrease in the starch content in tubers by 0.5%–9% compared to uninfected tubers (Blotskaya, 2000). It is noted that various viruses, for example, sweet potato leaf curl virus (SPLCV) and Tomato leaf curl New Delhi virus‐potato (ToLCNDV‐potato) lead to a significant decrease in the starch content in the sweet potato (Hou et al., 2020) and potato tubers (*Solanum tuberosum* L.) (Lal et al., 2021), respectively.

Potato starch possesses some unique physicochemical properties compared to starches from other sources (e.g., high phosphate content, lack of internal lipids, and proteins in the granules) (Burlingame et al., 2009; Romano et al., 2016). The properties of starch are affected by the ratio of amylose to amylopectin, granule size, phosphate content, and other parameters (Tang et al., 2002; Vasanthan et al., 1999). It is noted that among the effects of ToLCNDV‐potato on the potato plant, it reduces the content of starch and amylose (Lal et al., 2021). However, there are no studies on the properties of starch obtained from potato tubers infected with PVY, PVS, and PVM. Therefore, this study will systematically reveal the structure and physicochemical properties of potato starch isolated from tubers infected by PVY, PVS, and PVM, and the obtained results of this study hope to provide a basis for potato cultivation and potato starch processing and utilization.

## 2 MATERIALS AND METHODS

### 2.1 Samples and material

Samples of Artemis potato tubers clones naturally infected with PVM, PVY, and PVS, as well as virus‐free tuber clones, were provided by the Department of Biology, Plant Protection and Quarantine of S. Seifullin Kazakh Agrotechnical University. Potato tubers of the Artemis variety (originator – AGRICO U.A., Holland; second early maturity, average dry matter content 20.4%) were selected from Agrofirma Rodina LLP, Tselinograd district, Akmola region. After selection, 90 labeled tubers were germinated in the laboratory to obtain sprouts, which were tested by ELISA for the presence of viruses. To avoid reinfection with viruses, the tested monoinfected and virus‐free germinated tubers of potato plants were grown in separate pots under isolated phytotron conditions. To confirm the presence or absence of virus infection in potato clones grown in the phytotron, their leaf samples were again checked by RT‐PCR. The sample size of tuber samples for further research was 30 pcs.

### 2.2 Diagnosis of potato viruses

#### 2.2.1 ELISA testing

The DAS ELISA was used according to the attached instructions using 96‐well polystyrene tablets “Medpolymer.” The ELISA results were considered using a vertical light flow spectrophotometer (StatFax 4200, USA) at a wavelength of 492 nm. When carrying out this variant of ELISA, Russian commercial diagnostic kits of ELISA to determine potato viruses (PVX, PVY, PVS, PVM, and PLRV) of the Russian Potato Research Centre named after A.G. Lorkh were used (Simakov et al., 2000).

#### 2.2.2 PCR detection

Potato clone samples by RT‐PCR for the presence of potato viruses, PVY, PVS, PVM, PVX, and PLRV, with standard methods were studied (Dunaeva et al., 2011). Evaluation of potato samples for virus carrying with classical RT‐PCR in the plant biotechnology laboratory of the Department of Biology, Plant Protection and Quarantine of S. Seifullin Kazakh Agro Technical University was conducted. RNA from the NK‐Agro kit samples was isolated (Agrodiagnostika, Russia). To analyze the content of pathogens of potato viruses, reagent kits for reverse transcription of RNA and PCR amplification of cDNA of phytopathogenic viruses were used (Forez format, manufactured by Agrodiagnostics LLC, Russia). PCR reaction was carried out on a Thermal Cycler T1000 Toughch (Bio‐Rad, USA). When setting up RT‐PCR, samples of the same mass and volume were examined.

### 2.3 Starch isolation

Starch was extracted from fresh potato tubers by repeated washing with deionized water at room temperature, according to Richter et al. (1968).

### 2.4 Dry matter and starch content

Dry matter (Thermo‐ventilated oven at 105°C) and starch content were determined by the Evers polarimetric method.

### 2.5 Microscopic observation

#### 2.5.1 Scanning electron microscopy

Scanning electron microscopy (SEM) micrographs were taken using a scanning electron microscope (JSM‐6360LV, JEOL, Japan). A starch sample adhered to an SEM stub using double‐sided adhesive conductive tape and coated with a thin layer of gold to make the sample conductive. The mounted sample was then placed on the SEM stage, and images were digitally captured at the accelerating voltage of 10 kV.

#### 2.5.2 Polarized light microscopy

The starch sample was suspended in a 1:1 glycerol solution (glycerol/H2O, V/V) and was observed using light microscopy (DMBA400, Motic China Group Co., Ltd, Guangzhou, China) with the polarized light filter at 40‐time magnification.

### 2.6 Particle size distribution

The particle size distribution was measured using a laser light‐scattering particle size analyzer (Mastersizer 2000, Malvern Instruments Ltd., England). The starch was placed in water and analyzed automatically by laser diffraction.

### 2.7 Chemical characterization of the starches

Amylose levels were determined using the Juliano (1971) method. The glucose formed is then estimated calorimetrically. From the total starch content, the amylopectin fraction is determined by difference. Replicate samples (*n* = 4–6) were analyzed.

Starch moisture contents were determined by drying weighed amounts of starch in predried aluminum dishes in an air oven at 103°C to reach constant weight (Roder et al., 2009). Protein contents of duplicate samples were estimated from total nitrogen measurements (N 6.25) determined by a micro‐Kjeldahl method.

### 2.8 Thermal properties

The thermodynamic properties of starches were determined using differential scanning calorimetry DSC 1/200 W (Mettler Toledo, USA). Samples of starch (approximately 10.0 mg dry weight) were weighed directly into an aluminum crucible (Mettler, ME‐51119872), and distilled water was added in a 1:3 ratio. The crucible was sealed and equilibrated for 1 h before analysis. An empty hermetically sealed crucible was used as a reference. Then, the samples were heated from 30°C to 170°C at a rate of 10°C/min. As a result, the initial temperature (Tn), peak temperature (Tn), final temperature (Tk), and enthalpy (ΔH) were determined. The range was calculated by subtracting the initial temperature from the final temperature.

### 2.9 X‐ray diffraction (XRD)

Samples were analyzed using an X‐ray diffractometer (D8, Bruker, Germany), equipped with a copper tube at 40 kV. The scanning range of the diffraction angle (2θ) was from 4° to 60° at a speed of 6°/min, with a step size of 0.02°. The degree of crystallinity is calculated by Jade software (Jade 5.0).

### 2.10 Statistical analysis

All the experiments were the average of at least two replicates. The data were statistically analyzed using the SPSS 16.0 statistical package and expressed as mean value ± standard deviation. Statistical significance was established at *p* < .05.

## 3 RESULTS AND DISCUSSION

### 3.1 Diagnosis of potato viruses

In the first research stage, samples were taken from the potato tuber sprouts. To obtain healthy and monoinfected clones, a DAS‐ELISA test was conducted to identify the PVY, PVX, PVS, PVM, and PLRV.

The ELISA testing results of 90 samples showed a positive reaction mainly on PVY and its complexes with other viruses (PVS, PVM, and PLRV). No PLRV and PVA were detected. Artemis sample No. 65 showed a positive reaction to PVM (0.217 OD compared with 0.023 OD of negative control), Artemis sample No. 34 on PVS (0.845 OD compared to 0.021 OD of negative control), samples Artemis No. 1 and Artemis No. 13 are 0.534 and 0.458 OD compared to 0.008 OD, respectively. The ELISA optical density of Artemis sample No. 65 was at the same level as other viruses' negative control.

**FIGURE 1:**
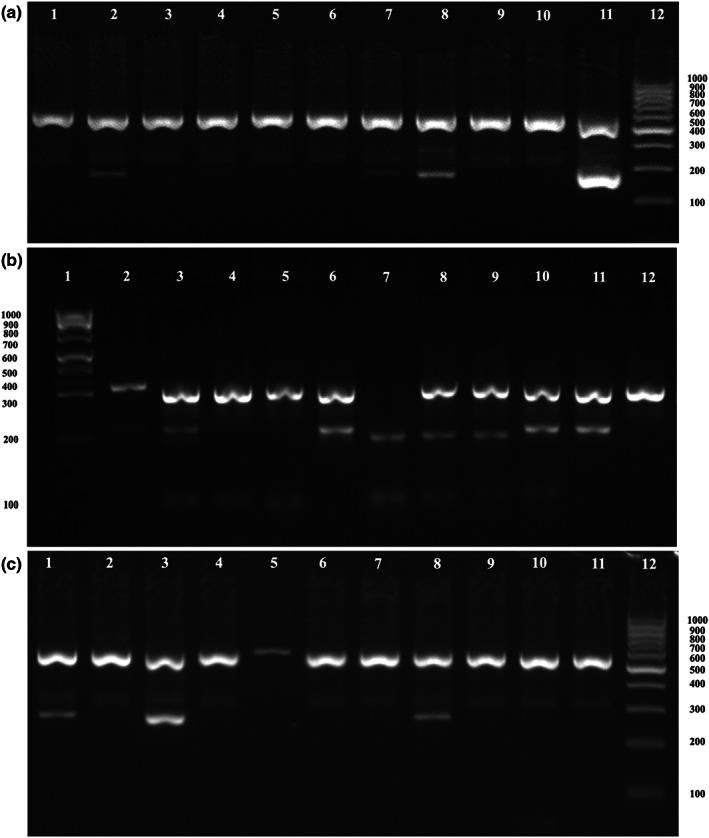
Electropherograms of potato sample PCR products, tested for PVM, PVS, and PVY in 1,5% agarose gel. (а) 1—Artemis No. 1; 2—Artemis No. 7; 3—Artemis No. 9; 4—Artemis No. 13; 5—Artemis No. 15; 6—Artemis No. 27; 7—Artemis No. 34; 8—Artemis No. 65; 9—Artemis No. 67; 10—Negative control; 11—Positive control; 12—Gene Ruler 100 bp DNA Ladder (Gene Ruler.). PCR product size—160 bp; (b) 1—Gene Ruler; 2—Artemis No. 1; 3—Artemis No. 3; 4—Artemis No. 13; 5—Artemis No. 65; 6—Artemis No. 14; 7—Artemis No. 20; 8—Artemis No. 23; 9—Artemis No. 28; 10—Artemis No. 34; 11—Positive control; 12—Negative control. PCR product size—278 bp; (с) 1—Positive control; 2—Negative control 3—Artemis No. 1; 4—Artemis No. 3; 5—Artemis No. 6; 4—Artemis No. 7; 5—Artemis No. 8; 6—Artemis No. 9; 7—Artemis No. 12; 8—Artemis No.13; 9—Artemis No. 34; 10—Artemis No. 65; 11—Artemis No. 67; 12—Gene Ruler. PCR product size—241 bp. Internal control, size—560 b.p.

To confirm the monoinfection and virus‐free potato clones, the samples were tested by RT‐PCR. Figure 1 shows the separation of PCR products of the studied potato samples infected with viruses: PVY, PVS, and PVM.

As a result of PCR, several monoinfected samples were identified and selected for further studies: PVM – Artemis No. 65 with a molecular weight of the PCR product of 160 bp (Figure 1a), PVS – Artemis No. 34 (278 bp) (Figure 1b) and two samples infected with PVY: Artemis No. 1 and Artemis No. 13 (Figure 1с). In addition, the Artemis No. 67 sample also confirmed the absence of viruses with PCR (Figure 1a–с).

### 3.2 Chemical properties and morphological characteristics of starch

As noted before, researchers found a decrease in the starch content in the tubers of potatoes infected with the virus (Blotskaya, 2000; Hou et al., 2020; Lal et al., 2021; Zhao et al., 2022; Amelyushkina, 2018; Rusetsky, 2006). The dry matter of tubers and starch content decrease in tubers of potato plants infected by PVM, PVS, and PVY. For example, compared with the tubers of virus‐free plants, the amount of starch in tubers infected by PVY decreased by 6.31%, PVM by 0.96%, and PVS by 0.04%. After this, starch samples are referred to by the name of the viruses that infect the plant, and starch isolated from tubers of healthy plants is referred to as virus free.

PVM, PVY, and PVS samples exhibit higher amylose content than the virus‐free control sample; the highest amount of amylose (22.01%) was noted in PVY starch. According to Lal et al. (2021), ToLCNDV‐potato infection caused changes to amylose content in starch, probably due to the effect of viruses on the biochemical and physiological process of starch accumulation in tubers, similar to the procedures described by Zhao et al., 2022.

Ash content in all samples correlates with the phosphate content. PVY and PVM increased ash content and phosphates in starch, while PVS slightly reduced it compared with the virus‐free sample.

**FIGURE 2:**
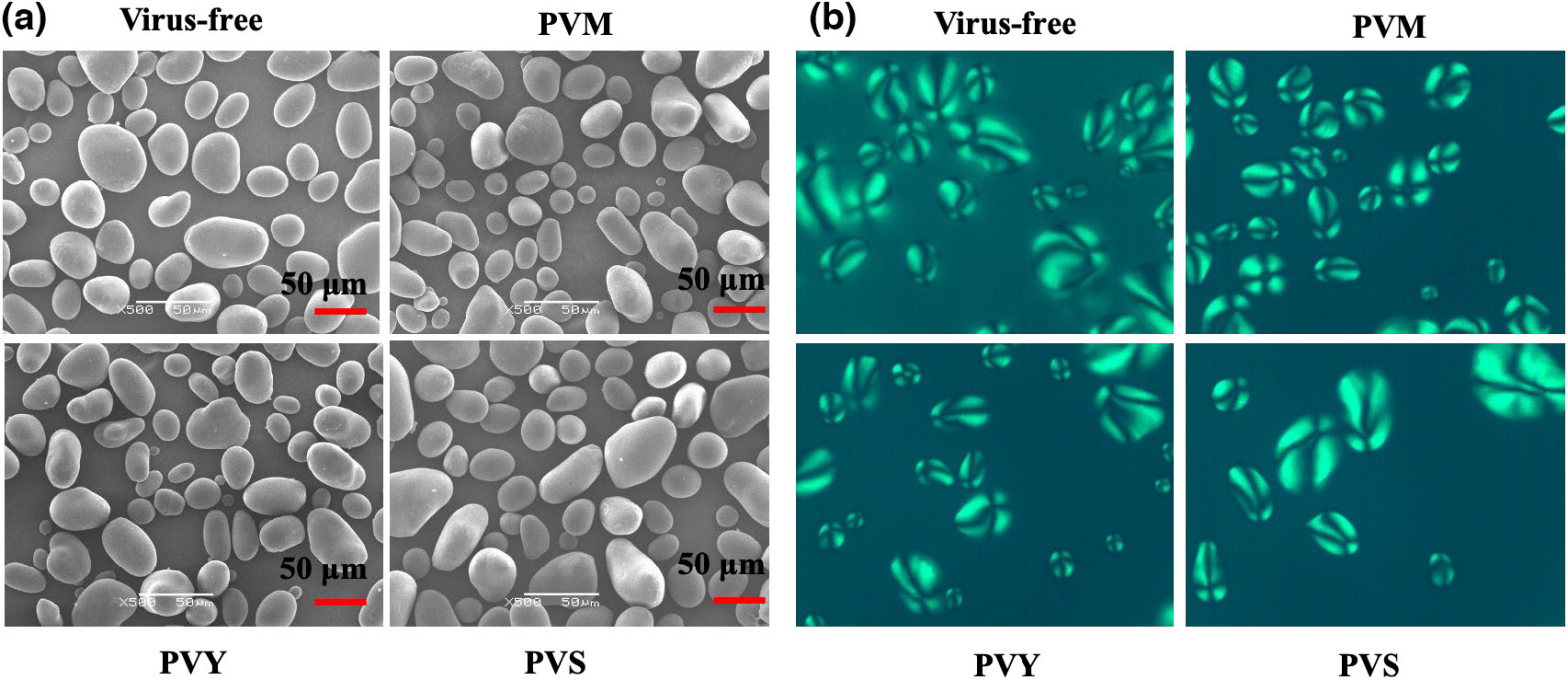
SEM (a) and PLM (b) of potato starch granule after virus‐free and infestation with PVM, PVY, and PVS viruses.

Morphological studies of potato starch samples are presented in Table 1 and Figure 2, and there is a difference in the size of granules with a spherical–oval shape. Table 1 shows the change in the size of potato starch granules depending on the infection of tubers with various viruses. The average diameter of virus‐free samples’ starch granules was 45.22 μm. Starch isolated from PVS‐infected potato tubers had larger granules (46.47 μm) than the virus‐free sample. In comparison, PVM and PVY samples were significantly smaller in diameter, 40.41 μm and 39.97 μm, respectively.

**TABLE. 1:** Chemical properties of «Artemis» variety potato tubers infected viruses PVM, PVY, and PVS and its starch granules.

Sample	Dry matter tuber (% FW)	Starch tuber (% DM)	Amylose (%)	Starch ash (%)	Phosphates starch (%)	Starch diameter (μm)
Virus free	25.40 ± 0.33	70.79 ± 0.31	18.87 ± 1.43	0.51 ± 0.01	0.0672 ± 0.004^c.d^	45.22 ± 0.06^а^
PVM	25.20 ± 0.05	70.11 ± 0.14	20.97 ± 1.73	0.63 ± 0.02	0.0744 ± 0.001^a^	40.41 ± 0.03^f^
PVY	24.26 ± 0.17	66.32 ± 0.48	22.01 ± 1.06	0.65 ± 0.04	0.0781 ± 0.001^b.c^	39.97 ± 0.07^b^
PVS	25.31 ± 0.08	70.76 ± 0.86	19.50 ± 1.56	0.49 ± 0.01	0.0638 ± 0.003^e^	46.47 ± 0.02^d^

*Note:* Values are means ± SD. Values with different superscripts within a column are significantly different (*p* < .05).

Even though the variety of sizes and shapes of starch grains is of various origins, the internal organization of these grains in starch is practically the same. Starch macromolecules are ordered in amorphous and semicrystalline parts (Jenkins & Donald, 1995).

Double helixes are perpendicular to the growth rings and closer to the surface of the starch grains than the center. The circular orientation is due to the birefringence structure, which can be fixed using crossed polarizers. All granules exhibit a polarizing cross.

### 3.3 X‐ray crystallization of native and infected starches

**FIGURE 3:**
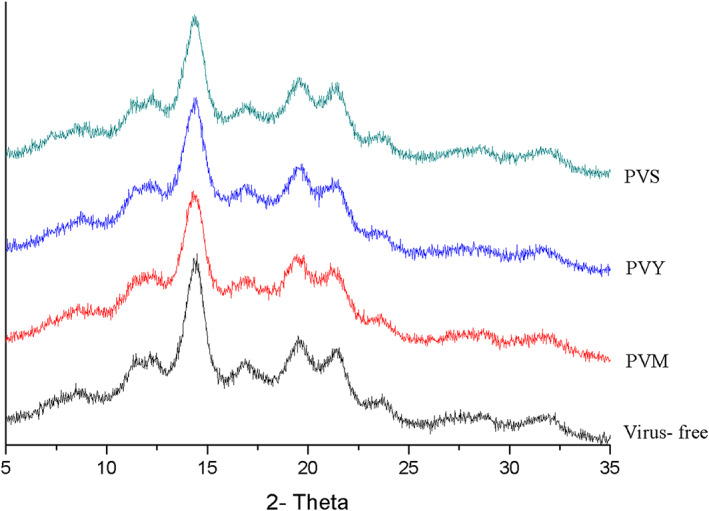
XRD pattern of potato tubers infected viruses PVM, PVY, PVS, and its starch granules.

X‐ray diffraction patterns of starch isolated from clones of Artemis potato tubers naturally infected with PVM, PVY, and PVS and starch clones of virus‐free tubers are shown in Figure 3. The studied starch samples present a typical B‐type crystal structure, i.e., diffraction peaks at diffraction angles of 15°, 17°, 20°, and 23° (2θ), respectively (Figure 3), indicating that these viruses did not significantly change the starch crystallization pattern.

The degree of crystallinity of starch granules ranges from 37.22% to 38.46%. Although the data obtained in this work are within the range of values reported for potato starch by other authors, literature values for starch crystallinity vary from 15% to 45% (Alvani et al., 2011). Data received during the investigation of starches obtained from tubers infected by viruses show a decrease in crystallinity in all cases (37.22%–38.10%) compared to virus free (38.46%).

### 3.4 Differential scanning calorimetry

The thermal characteristics of native potato starches determined by the differential scanning calorimetry (DSC) are shown in Table 2. A summary of DSC results for the starches is presented in Table 2, which offers the values for gelatinization onset (To), peak (Tp), and conclusion (Tc) temperatures as well as gelatinization enthalpies (ΔH). То, Тр, and Тс of native virus‐free starch samples were 66.23, 69.38, and 73.98°С, respectively. PVM and PVY samples were observed to have an insignificant decrease in to, while the PVS sample had a slight increase compared to the virus‐free sample. The apparent reduction in gelatinization temperatures can be explained by the destruction of amylopectin chains and a decrease in overall crystallinity (Zhou et al., 2020). It is possible that the influence of PVM and PVY on the metabolic process of potato plants affected the crystal structure of starch granules, partially destroying them, and thereby enhancing granules' ability to swell. In addition, an increase in gelatinization enthalpy in starches isolated from virally infected tubers is observed (ΔH). ΔH increased from 5,44 J/g (virus‐free) to 8,17 J/g (PVY). This points to the conclusion that more energy is needed to gelatinize them.

**TABLE. 2:** XRD properties and thermal properties of «Artemis» variety potato tubers infected viruses PVM, PVY, and PVS and its starch granules.

Sample	XRD properties				Thermal properties			
	Diffraction angle intensity			Crystallinity	Тo (°C)	Тр (°C)	Тc (°C)	Δ*H* (J/g)
Virus free	1204	349.4	406.6	38.46%	66.23	69.38	73.98	5.44
PVM	1135	350	285	38.10%	65.75	69.07	73.99	5.68
PVY	1164	374	343	37.22%	65.06	68.28	73.53	8.17
PVS	1204	370	404	37.51%	66.34	69.27	74.05	7.36

*Note:* Values are means ± SD. Values with different superscripts within a column are significantly different (*p* < .05).

## 4 CONCLUSIONS

Infection of potato plants by PVM, PVY, and PVS leads to a decrease in the dry matter and starch content in tubers. Starch samples from potato tubers infected with PVM, PVY, and PVS had higher amylose content. Granules of starch isolated from potato tubers infected by PVS exhibit larger granules than starch granules isolated from tubers of healthy plants. In contrast, in the case of PVM and PVY infection, starch granules were significantly smaller in diameter. All starch samples had B‐type crystalline structure typical of potato starches, a decrease in the degree of crystallinity has been observed in all samples of starches obtained from the tubers of infected plants compared to starch isolated from virus‐free samples. A slight decrease in gelatinization temperature was noted for starch samples isolated from tubers infected by potato viruses PVM and PVY, and a slight increase in gelatinization temperature for starch samples isolated from tubers infected by PVS compared to starch isolated from tubers of healthy plants. In all samples of starch obtained from tubers of infected plants, an increase in the value of gelatinization enthalpy is observed. Thus, it can be concluded that damage to potato plants by PVY and PVM leads to a significant decrease in the quality of starch in tubers. At the same time, infection by PVS had practically no considerable effect.

## AUTHOR CONTRIBUTIONS

**Gulnazym Ospankulova:** Formal analysis (equal); funding acquisition (equal); project administration (equal); resources (equal); supervision (equal); writing – original draft (equal). **Vadim Khassanov:** Investigation (equal). **Svetlana Kamanova:** Formal analysis (equal). **Dana Toimbayeva:** Methodology (equal). **Saule Saduakhasova:** Conceptualization (equal). **Berdibek Bulashev:** Data curation (equal). **Gulnar Aidarkhanova:** Validation (equal). **Yernaz Yermekov:** Software (equal). **Linara Murat:** Data curation (equal). **Bakhyt Shaimenova:** Methodology (equal). **Marat Muratkhan:** Data curation (equal); formal analysis (equal); software (equal).

## FUNDING INFORMATION

This research was funded by the Science Committee of the Ministry of Education and Science of the Republic of Kazakhstan, grant number AP08857439.

## CONFLICT OF INTEREST STATEMENT

The authors declare that they do not have any conflict of interest.

## ETHICAL APPROVAL

This study does not involve any human or animal testing.

## INFORMED CONSENT

Written informed consent was obtained from all study participants.

## Data Availability

The data supporting this study are available on request from the corresponding author.
